# Supplementary figures and images for: Dysregulation of the hippocampal neuronal network by LGI1 auto-antibodies
Source: PLoS One. 2022 Aug 19;17(8):e0272277. doi: 10.1371/journal.pone.0272277 (PMC9390894; doi:10.1371/journal.pone.0272277)

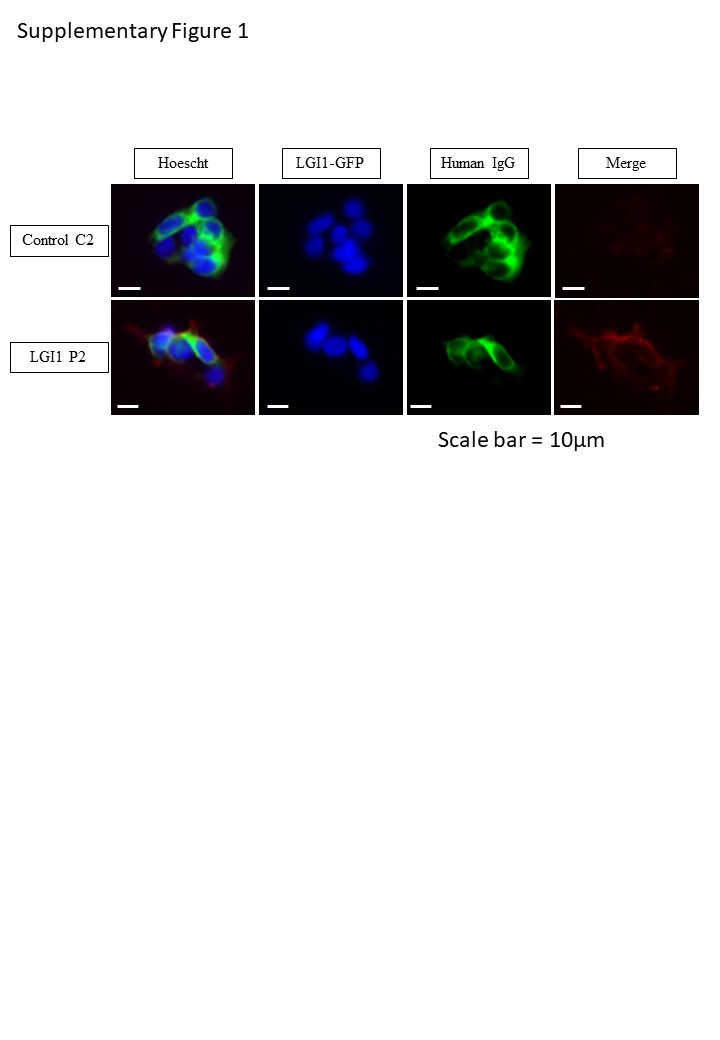

Supplement: S1 Fig — HEK293T cells were transfected with LGI1-GFP and ADAM22 plasmids. Purified IgG from LGI1 LE patient 2 are colocalized with LGI1-GFP signal at the cell surface of HEK293T transfected cells while no signal was found in cells treated with purified IgG from healthy subject. Scale bar = 20μm. (TIF) [file pone.0272277.s001.TIF]

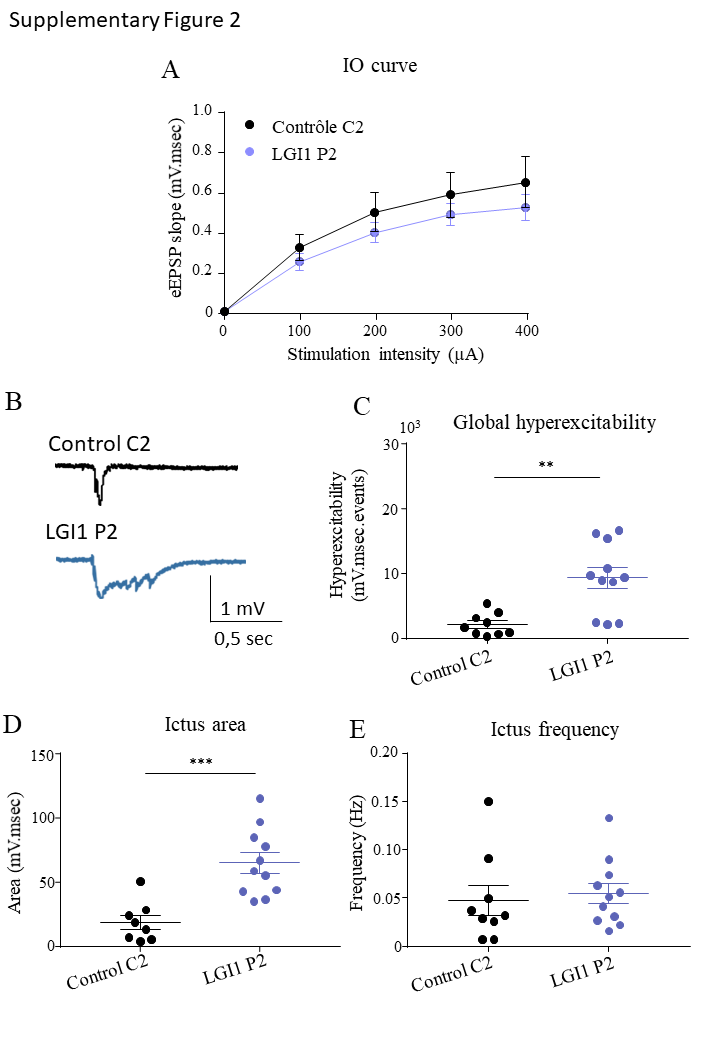

Supplement: S2 Fig — LFP were recorded in CA1 region of acute hippocampal slices from mice infused for 7 days with control C1 IgG, LGI1 P2. (A) Increasing stimulations intensity were delivered to Schaffer collaterals and the slope of eEPSP in CA1 area were plotted. No effect of the infusion of control C2 IgG, LGI1 P2 on the IO curve was detected (two-way ANOVA test, p = 0,27; nCtrl = 12; nLGI1 = 14). (B) Examples of ictus recorded in slices infused with control C2 IgG, LGI1 P2 IgG. (C) The global hyperexcitability of the neuronal network was significantly increased on slices infused with LGI1 P2 IgG compared to slices infused with control C2 IgG (** for p = 0,0023, Mann Whitney test, nC2 = 9 souris nP2 = 11 mice). (D) The ictus area was significantly increased between slices infused with LGI1 P2 IgG and slices infused with control C2 IgG (*** for p = 0,0004; nC2 = 9 nP2 = 11 mice). (E) The ictus frequency was not different between LGI1 P2 IgG, control C2 IgG (Mann Whitney test: p = 0,44; nC1 = 9, nP1 = 11 mice). Data are represented as mean ± SEM. (TIF) [file pone.0272277.s002.TIF]

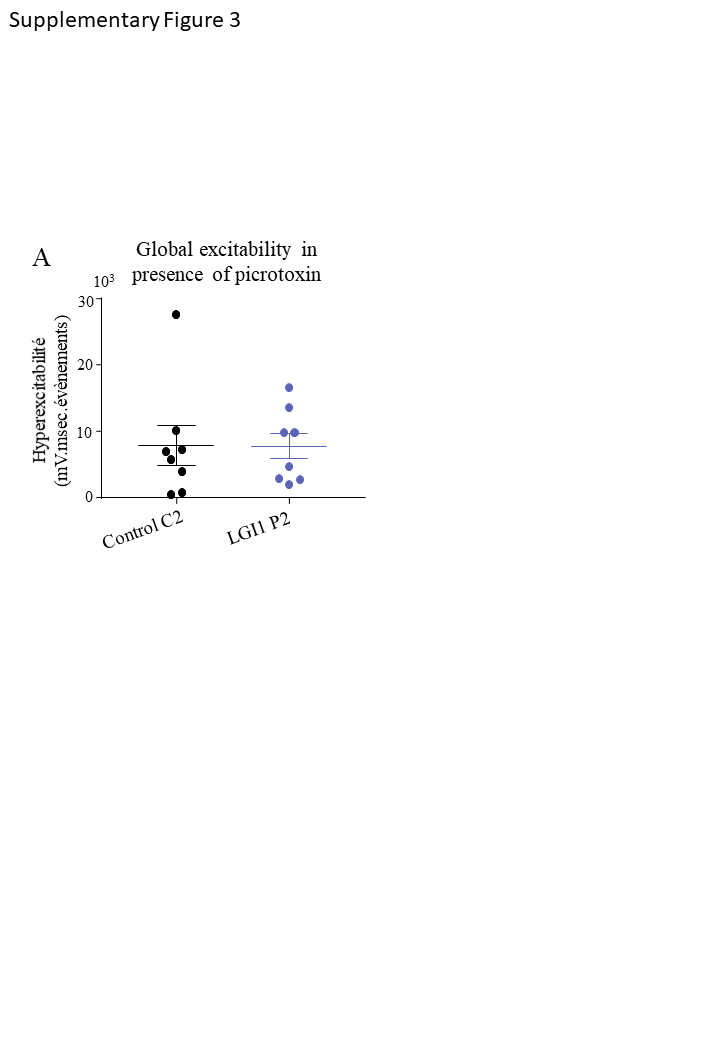

Supplement: S3 Fig — (A) LFP in presence of 4-AP (40μM) and picrotoxin (100μM) were recorded in CA1 area of acute hippocampal slices from mice infused for 7 days with control C2 or LGI1 P2 IgG. No difference was found in the global hyperexcitability between control C2 IgG and LGI1 P2 IgG infused neuronal network when recordings were performed in presence of picrotoxine (Mann-Whitney test, p = 0,80; nC2 = 8; nP2 = 8 mice). Data are represented as mean ± SEM. (TIF) [file pone.0272277.s003.TIF]
